# Longevity of immune response after a single dose of typhoid conjugate vaccine against *Salmonella* Typhi among children in Hyderabad, Pakistan

**DOI:** 10.1016/j.ijid.2024.107187

**Published:** 2024-10

**Authors:** Farah Naz Qamar, Sonia Qureshi, Zoya Haq, Tahir Yousafzai, Ibtisam Qazi, Seema Irfan, Najeeha Iqbal, Zohra Amalik, Aneeta Hotwani, Qumber Ali, Irum Fatima, Najeeb Rahman, Alice S. Carter, Jessica C. Seidman

**Affiliations:** 1 Department of Pediatrics and Child Health, Aga Khan University Hospital, Karachi, Pakistan; 2 Liaquat National Medical College, Karachi, Pakistan; 3 Department of Microbiology, Aga Khan University Hospital, Karachi, Pakistan; 4 Sabin Vaccine Institute, Washington, USA

**Keywords:** Immune response, Typhoid conjugate vaccine, Children

## Abstract

**Objectives:**

Typhoid remains a persistent contributor to childhood morbidity in communities lacking sanitation infrastructure. Typhoid conjugate vaccine (TCV) is effective in reducing disease risk in vaccinees; however, the duration of protection is unknown. This study measured the longevity of immune response to TCV in children aged under 10 years in Hyderabad, Pakistan, where an outbreak of extensively drug-resistant typhoid has been ongoing.

**Methods:**

A subset of children who received the TCV as part of the outbreak response were enrolled purposively from March 2018 to February 2019. The participants were followed up until January 2023. Blood samples were taken at baseline, 4-6 weeks, 6 months, and annually 1-4 years after vaccination to measure anti-Vi immunoglobulin (Ig) G levels using enzyme-linked immunosorbent assay. Active phone-based surveillance was performed to identify breakthrough infections. Blood culture was offered to any child with a history of fever ≥3 days within the last 7 days. A total of 81 children received a second dose of TCV in November 2019 during a catch-up campaign organized by the Sindh government.

**Results:**

Nearly all participants seroconverted (802 of 837; 95.8%) at 4-6 weeks after vaccination. A total of 4 years after vaccination, 438 of 579 (75.6%) participants remained above the seroconversion threshold. The geometric mean titer (U/mL) of anti-Vi IgG at 4-6 weeks was 832.6 (95% confidence interval [CI]: 768.0-902.6); at 4 years after vaccination, the geometric mean titers in children aged 6 months to 2 years (12.6, [95% CI: 9.8-16.3]) and >2-5 years (40.1, [95% CI: 34.4-46.6]) were lower than in children aged >5-10 years (71.1, [95% CI: 59.5-85.0]). During 4 years of follow-up, nine children had culture-confirmed *Salmonella* Typhi infection; these infections occurred after a median duration of 3.4 years. All enteric fever cases seroconverted at 4-6 weeks after vaccination and seven (70.0%) remained seroconverted 4 years after vaccination.

**Conclusions:**

We observed 95.8% seroconversion after a single dose of TCV. There was a decay in anti-Vi IgG titers, and, at 4 years, approximately 75.6% remained seroconverted. There was a faster decay in children aged ≤2 years. Breakthrough infections were documented after a median 3.4 years after vaccination.

## Highlights

• *Salmonella* Typhi can cause a potentially life-threatening infection, typhoid fever*.*
• Typhoid conjugate vaccine (TCV) has been established as a safe, well-tolerated, and effective vaccine.
• A high seroconversion was observed at 4-6 weeks after a single dose of TCV.
• A single dose of TCV provides durable immunogenicity.

## Introduction

The bacterium *Salmonella* Typhi can cause a potentially life-threatening infection, typhoid*.* Spread through contaminated food and water, typhoid affects an estimated 27 million people each year, causing 129,000-223,000 deaths worldwide [1]. Pakistan has an estimated typhoid incidence of 493.5 per 100,000 people per year [[2], [3], [4]], with unsafe water supply and poor sanitary conditions leading to large epidemics of this disease [5].

The widespread misuse of antibiotics in Pakistan due to their easy availability led to the emergence of an extensively drug-resistant (XDR) strain of typhoid fever [[6], [7], [8]]. This strain poses a threat to the effective management of typhoid because the strain is resistant to all commonly available antibiotics [9,10]. Since the first identification of the XDR isolate in November 2016, the XDR typhoid strain expanded rapidly to various districts of Sindh [[10], [11], [12]]. To address this outbreak of XDR typhoid, a large-scale immunization campaign using the World Health Organization (WHO)–recommended typhoid conjugate vaccine (TCV) was implemented in January 2018, targeting the most affected areas of Hyderabad city, i.e. Latifabad and Qasimabad [13]. After the vaccination campaign in Hyderabad, in 2019, Pakistan became the first country in the world to introduce the WHO-recommended TCV into its routine vaccination regimen through the Expanded Programme of Immunization [14].

TCV has been established as a safe, well-tolerated, and effective vaccine; however, there are limited data on the immunogenicity and long-term durability of response to TCV in community settings. A prototype TCV (Vi capsular polysaccharide bound to recombinant mutant *Pseudomonas aeruginosa* exoprotein A [Vi-rEPA]) given as a two-dose regimen in children 2-5 years of age showed a protective efficacy of 89% (95% confidence interval [CI]: 76.0-96.9%) over 46 months of follow-up [15]. In field evaluations and clinical trials, a single dose of a newer TCV was shown to induce a strong immunologic response in children as young as 6 months of age, a seroconversion frequency of 97-99%, and a protective efficacy of 82% in children aged 9 months to 16 years [[16], [17], [18], [19]]. We aimed to measure the serologic response using enzyme-linked immunosorbent assay (ELISA) anti-Vi immunoglobulin (Ig)G antibodies at several time points after immunization with TCV in a cohort of children vaccinated in the outbreak areas of Hyderabad, Pakistan.

## Methods

### Study design and setting

A prospective cohort study was conducted in Hyderabad, Pakistan from March 2018 to January 2023. We conducted a mass TCV campaign in the Qasimabad and Latifabad districts of Sindh, Pakistan, where a large number of XDR typhoid cases were reported. The vaccine used in the campaign was Typbar TCV (India), manufactured by Bharat Biotech Ltd. The population of these two districts is around 0.9 million, of which approximately 0.2 million are children between the ages of 6 months and 10 years [10,20].

### Participants

During the immunization drive in Hyderabad, around 207,000 children between the ages of 6 months to 10 years received a single intramuscular shot of TCV (21). We purposively enrolled 958 healthy children aged 6 months to 10 years at baseline and vaccinated with TCV during this campaign. Children with any acute illness or underlying chronic diseases were excluded. From the main cohort, 81 children received a second dose of TCV in November 2019 during a catch-up campaign organized by the government of Sindh.

### Procedure

Blood samples were collected for anti-Vi IgG antibodies at baseline (before vaccination) and at six time points (4-6 weeks, 6 months, 1 year, 2 years, 3 years, and 4 years) after vaccination. Data on socio-demographic characteristics, date of vaccination, and parents’ education were collected using a structured questionnaire at enrollment. Anthropometric measurements were collected (height/length [centimeters], weight [kilograms], and mid-upper arm circumference [centimeters]) using standard measuring scales and methodology by trained staff at baseline and annually 1-4 years after TCV vaccination. A vaccination card with the study enrollment number, date and time of vaccination, and the number of a 24-hour hotline for contact in case of any queries related to the vaccine was provided to each participant's caregiver after vaccination.

Active surveillance was conducted through monthly phone calls to identify cases of fever. During the phone calls, trained research staff asked the primary caregiver if the child had a history of fever for ≥3 days during the past month, if the child had been taken to any health care facility for treatment, or if any blood tests had been performed. If the child was ill, the study team visited the house to collect clinical illness details, any hospitalization or clinic/emergency visits, and the results of any laboratory investigations. Children who reported a persistent fever for ≥3 days with no clear focus of infection within the last 7 days of the phone call were offered blood culture from the nearest Aga Khan University satellite laboratory.

### Sample collection and testing

#### Blood sample collection and transportation

Approximately 3 mL of blood was collected from each enrolled participant by a trained phlebotomist using aseptic technique. Samples were transported under temperature control to the research laboratory in Matiari, Sindh, where serum was separated by centrifugation and transported to research laboratory in Karachi at 2-8°C on the same day. The serum was aliquoted and stored at −20°C until testing.

#### Laboratory assay for anti-Vi immunoglobulin G

The concentration of anti-Vi IgG antibodies was measured using the VaccZyme Human Anti-*Salmonella* Typhi Vi IgG Enzyme Immunoassay Kit (Binding Site Group Ltd., Birmingham, UK), following the manufacturer's instructions [21].

### Outcomes

The outcomes of interest were the seroconversion (four-fold rise in anti-Vi IgG titer) rate at each time point compared with baseline and the number of breakthrough infections during the follow-up study period.

### Data analysis

Geometric mean antibody titers (GMTs) and 95% CIs were calculated from the anti-Vi IgG (U/mL) concentrations at each time point. GMTs were stratified by age (6 months to 2 years, >2-5 years, and >5-10 years). The threshold for seroconversion was defined as a four-fold rise in anti-Vi IgG antibodies above the baseline antibody level; seroconversion was calculated for each time point. Participants who received an additional dose of TCV during the government immunization campaign were excluded from the primary seroconversion, and GMT analyses for the time points that were more than 4 weeks after the 2^nd^ dose was received: seven of 21 children who received second dose of TCV at approximately 10 months after a first dose were excluded from the analysis of 1 year time point, and all 81 children who received a second dose during the study were excluded from the analysis of the 2-4 year time points to avoid any bias.

Anthropometric measurements were obtained from all participants at baseline. Weight for length (WLZ), weight for height (WHZ), and body mass index for age (BAZ) Z-scores were calculated for children aged 6 months to 2 years, >2-5 years, and >5-10 years using the WHO Anthro software. The nutritional status was divided in two categories as well-nourished (WHZ/WLZ/BAZ score ≥−2 to ≤+6) and malnourished (WHZ/WLZ/BAZ score ≤−6 to <−2) and excluding children whose WHZ/WLZ/BAZ scores were greater than +6 or less than −6 SDs.

Data were analyzed using STATA version 16.0. Frequencies with percentages were reported for categorical variables such as gender, seroconversion, nutritional status, etc. For continuous variables such as age (years), the mean/median and SD/interquartile range (IQR) were reported. The association between GMTs and age groups was assessed using analysis of variance on logarithmically (Log_10_) transformed data. Subsequently, the Tukey test was used for *post hoc* pairwise comparisons with a *P*-value adjustment for multiple comparisons. A chi-square test was applied for assessing the relationship between seroconversion and age categories and *P*-values for *post hoc* pairwise comparisons were adjusted with the Bonferroni method for multiple comparisons. The Kruskal–Wallis test was conducted to analyze the association between the median anti-Vi IgG levels and age groups. An independent sample *t*-test was used to analyze the association between median antibody titers and seroconversion. A *P* ≤0.05 was considered statistically significant.

## Results

A total of 958 children were enrolled and administered a single dose of TCV from March 2018 to February 2019. At baseline, blood samples from 958 (100%) children were collected for ELISA and anti-Vi IgG level analysis.

**Figure 1:**
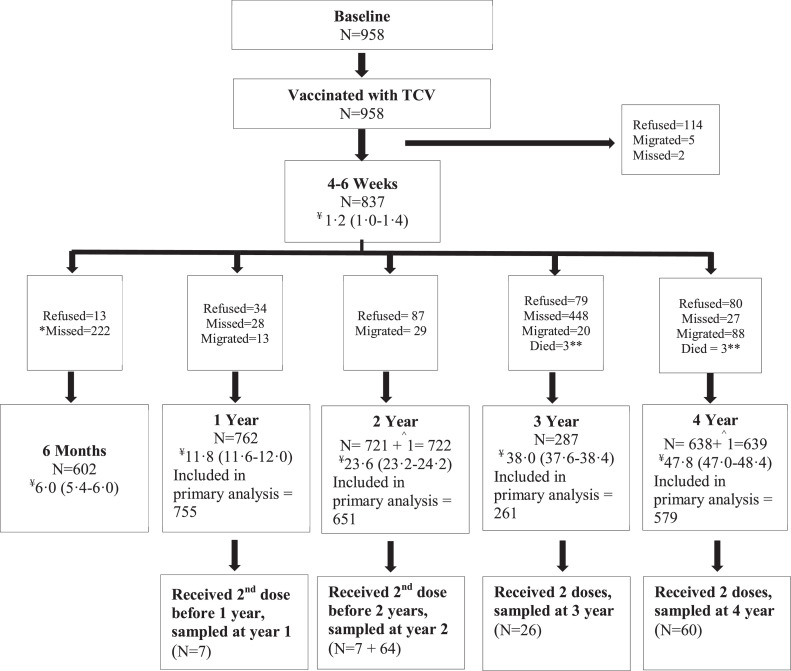
Study flow. TCV, typhoid conjugate vaccine. *Children were missed because a 6-month time point was added later in the study. **A total of 3 children died during 4 years of follow-up before the 3-year time point. ^One child refused sampling for 4-6–week time point but provided sample at the 2- and 4-year time points. ¥Median (interquartile range) time of sampling in months at each time point.

After vaccination, 837 children provided a sample at 4-6 weeks, 602 at 6 months, 762 at 1 year, 722 at 2 years, 287 at 3 years, and 639 at 4 years. Figure 1 depicts the details of participant enrollments, follow-ups, and median (IQR) sampling time at each time point, as well as reasons for dropout and exclusion. The median age of the study participants at enrollment was 3.5 (IQR: 1.9-5.3) years (Table 1). The majority of participants were enrolled from the Qasimabad area (568 of 958; 59.3%), and there was an even gender split (501 of 958; 52.3%). Most of the children (693 of 855; 81.1%) were well-nourished.

**Table 1:** Demographic characteristics of study participants at baseline.

Variables	Categories	N = 958 n (%)
Age (years), median (interquartile range)		3.5 (1.9-5.3)
Age	6 months to 2 years	247 (25.8)
	>2-5 years	461 (48.1)
	>5-10 years	250 (26.1)
Gender	Male	501 (52.3)
Area of residence	Qasimabad	568 (59.3)
	Latifabad	390 (40.7)
Fathers education	No formal education or religious education only	248 (24.9)
	Primary/secondary	329 (34.3)
	Higher secondary or above	381 (39.8)
Mothers education	No formal education or religious education only	404 (42.2)
	Primary/secondary	262 (27.3)
	Higher secondary or above	292 (30.5)
Height (cm), mean ± SD		94.3 ± 17.5
Weight (cm), mean ± SD		13.9 ± 5.3
Mid upper arm circumference (cm), mean ± SD		14.8 ± 2.4
Body mass index (kg/m^2^), mean ± SD (N = 958)		18.1 ± 6.0
Nutritional status (N = 855)	Well nourished	693 (81.1)
	(WHZ/WLZ or BAZ score ≥−2 to ≤+6)	
	Malnourished	162 (18.9)
	(WHZ/WLZ or BAZ score ≤−6 to <−2)	

BAZ, body mass index for age WHZ, weight for height Z-score; WLZ, weight for length Z-score.

Seroconversion was observed in nearly all participants 802 of 837 (95.8%) at 4-6 weeks after a single dose of TCV. Most participants remained above the seroconversion threshold during follow-up, with 438 of 579 (75.6%) remaining seroconverted 4 years after vaccination.

The frequency of seroconversion at 4-6 weeks was higher in younger children aged ≤2 years (99.5%) than in older children (>2-5 years, 95.8% and >5-10 years, 92.5%) (Table 2, Table S1). However, during follow-up, the antibody decay in younger children was faster, and only 63.1% children in the ≤2 years age group remained seroconverted at the 4-year time point compared with children aged >2-5 years (78.2%) and >5-10 years (82.7%).

**Table 2:** Sero-conversion and GMT of study participants at different time points by enrollment age.

Age at vaccination	GMT									Seroconversion				
	Overall		6 months to 2 years		>2-5 years		>5-10 years		a*P*-value	Overall	6 months to 2 years	>2-5 years	>5-10 years	a*P*-value
Timepoints	N	GMT	N	GMT	N	GMT	N	GMT		%	%	%	%	
		(95% CI)		(95% CI)		(95% CI)		(95% CI)		(95% CI)	(95% CI)	(95% CI)	(95% CI)	
**Baseline**	958	2.6	247	1.7	461	3.1	250	2.7	<0.001	..	..	..	..	..
		(2.4, 2.8)		(1.5 - 1.9)		(2.8 - 3.6)		(2.2, 3.2)		..	..	..	..	..
**4-6 weeks**	837	832.6	207	662.0	402	958.4	228	800.1	0.001	95.8	99.5	95.8	92.5	0.001
		(768.0, 902.6)		(564.1 - 776.8)		(861.3 - 1066.5)		(671.7, 952.9)		(94.2-97.0)	(96.6, 99.9)	(93.3, 97.4)	(88.3, 95.3)	
**6 months**	602	115.2	145	57.8	251	135.9	206	152.9	<0.001	89.9	91.7	88.8	89.8	0.66
		(104.8, 126.5)		(45.9 - 72.9)		(120.8 - 152.9)		(132.4, 176.6)		(87.2, 92.0)	(86.0, 95.2)	(84.3, 92.2)	(84.9, 93.3)	
**1 year**	755	54.7	187	20.6	361	69.3	207	87.4	<0.001	82.9	74.3	85.3	86.5	0.001
		(49.5, 60.4)		(16.6 - 25.5)		(61.3 - 78.4)		(74.3 - 102.9)		(80.1, 85.4)	(67.6, 80.1)	(81.3, 88.6)	(81.1, 90.5)	
**2 years**	651	47.0	172	14.6	307	64.9	172	84.8	<0.001	80.2	68.0	85.0	83.7	<0.001
		(41.7, 52.9)		(11.5 - 18.5)		(55.8 - 75.4)		(70.6 – 101.8)		(76.9, 83.1)	(60.7, 74.6)	(80.6, 88.6)	(77.4, 88.5)	
**3 years**	261	33.5	80	10.1	92	46.0	89	71.0	<0.001	77.8	66.3	82.6	83.1	0.012
		(28.4, 39.5)		(7.7 - 13.2)		(36.6 - 57.7)		(58.1, 86.8)		(72.3, 82.4)	(55.2, 75.8)	(73.4, 89.1)	(73.9, 89.6)	
**4 years**	579	35.2	149	12.6	262	40.1	168	71.1	<0.001	75.6	63.1	78.2	82.7	<0.001
		(31.2, 39.6)		(9.8 - 16.3)		(34.4 - 46.6)		(59.5, 85.0)		(72.0-79.0)	(55.0, 70.5)	(72.8, 82.8)	(76.2, 87.7)	

CI, confidence interval; GMT, geometric mean antibody titers; N, number of children sampled; (..), not applicable.

a Association between geometric mean antibody titers and age were determined by analysis of variance and association between seroconversion and age were assessed by chi-square test.

**Figure 2:**
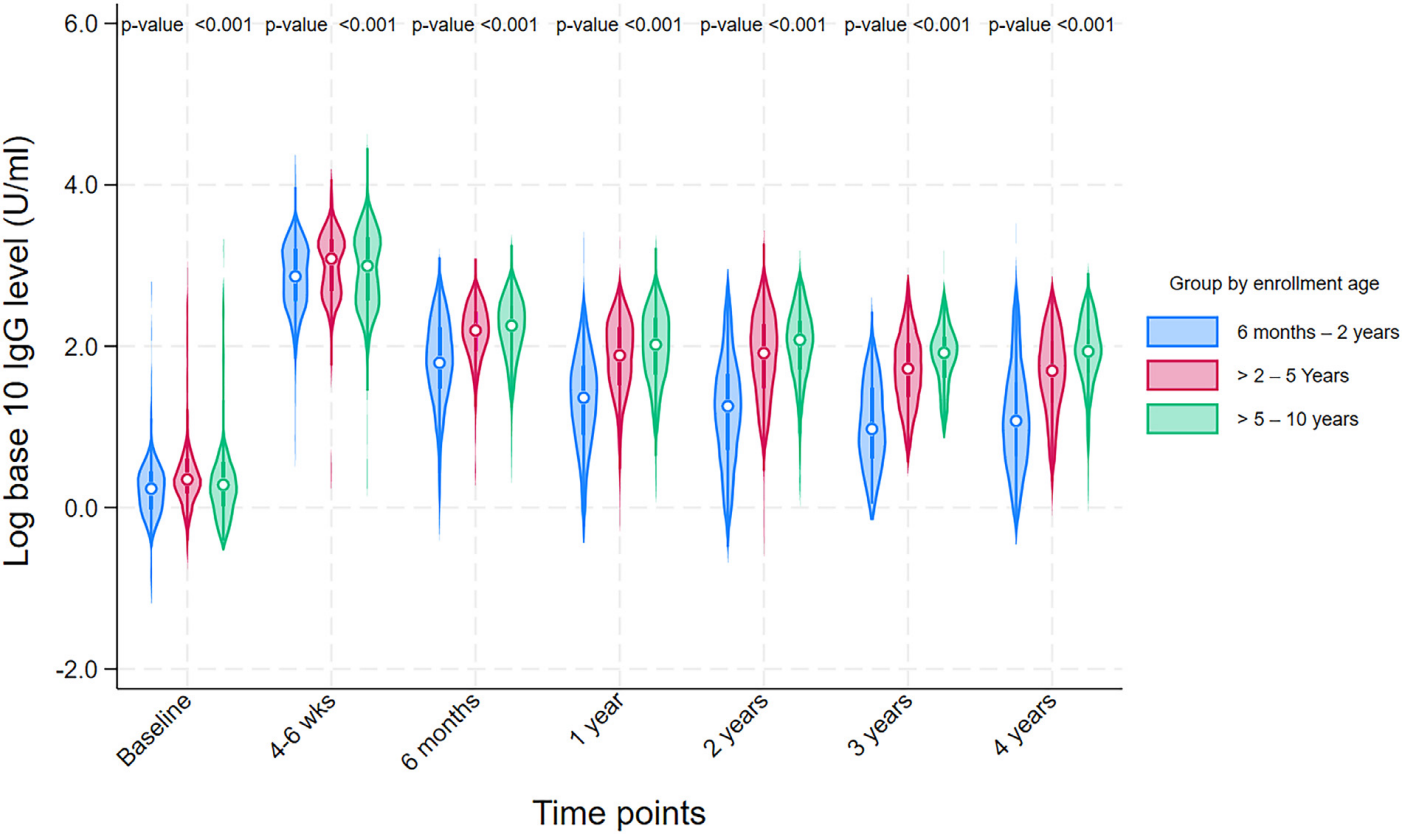
Age-stratified median (interquartile range) level of anti-Vi IgG among younger and older children. Ig, immunoglobulin. Kruskal–Wallis test was applied to determine the association between median anti-Vi IgG levels and enrollment age categories.

The anti-Vi IgG GMT (U/mL) peaked at 4-6 weeks in all children (662.0, 95% CI [564.1-776.8] in those aged ≤2 years; 958.4, 95% CI [861.3-1066.5] in those aged >2-5 years; 800.1, 95% CI [671.7, 952.9] in those aged >5-10 years) after vaccination and subsequently declined over the 4-year follow-up period (12.6, 95% CI [9.8-16.3] in those aged ≤2 years; 40.1, 95% CI [34.4-46.6] in those aged >2-5 years; 71.1, 95% CI [59.5, 85.0] in those aged >5-10 years) but remained elevated above baseline levels in all age groups (Table 2). Children aged ≤2 years had lower anti-Vi titers at each time point than children aged above 2 years (Figure 2, Table S2). Participants who did not seroconvert at 4-6 weeks already had high antibody titers at baseline and similar titers at the later post-vaccination time points compared with those who did seroconvert (Figure S1).

During 4 years of active surveillance, there were 174 reported episodes of fever from 140 children; blood cultures were performed for 60 participants, of whom 10 tested positive for enteric fever (*S*. Typhi = 9, *Salmonella* Paratyphi A = 1). The remaining 112 reported fever episodes did not occur within the preceding 7 days of the follow-up call, and two families refused blood culture testing. All participants who developed *S*. Typhi infections had seroconverted at the 4-6–week time point and received only a single dose of TCV. The median duration between vaccination and blood culture–confirmed infection with typhoid was 3.4 years (IQR: 2.4-3.8) (Table 3). The details of culture-confirmed cases are shown in Table S3A & S3B. Four of nine *S.* Typhi strains isolated were XDR and two were multi-drug–resistant. None of the cases developed any complications but four of nine children infected with *S*. Typhi required hospitalization. No mortality was observed due to typhoid fever; three children died during the study period (one due to pneumonia, one with brain tumor, and one due to accidental drug ingestion).

**Table 3:** Characteristics of culture-confirmed enteric fever cases (N = 10).

Variables		n (%)
Seroconversion at 4-6 weeks	No	0 (0.0)
	Yes	10 (100.0)
Serotype Isolated	*S*. Paratyphi	1 (10.0)
	*S*. Typhi	9 (90.0)
Drug susceptibility pattern of *S*. Typhi isolates (N = 9)	Multi-drug resistant	2 (22.2)
	Extensively drug resistant	4 (44.4)
	aNon–multi-drug resistant/extensively drug resistant	3 (33.3)
Antibody titers at the time of infection (U/mL) (geometric mean antibody titers [95% confidence interval])	..	24.3 (9.7-60.8)
Duration (years) between vaccination and infection (Median [interquartile range])	..	3.4 (2.4-3.8)

a Sensitive to chloramphenicol, co-trimoxazole, azithromycin, and imipenem.

**Figure 3:**
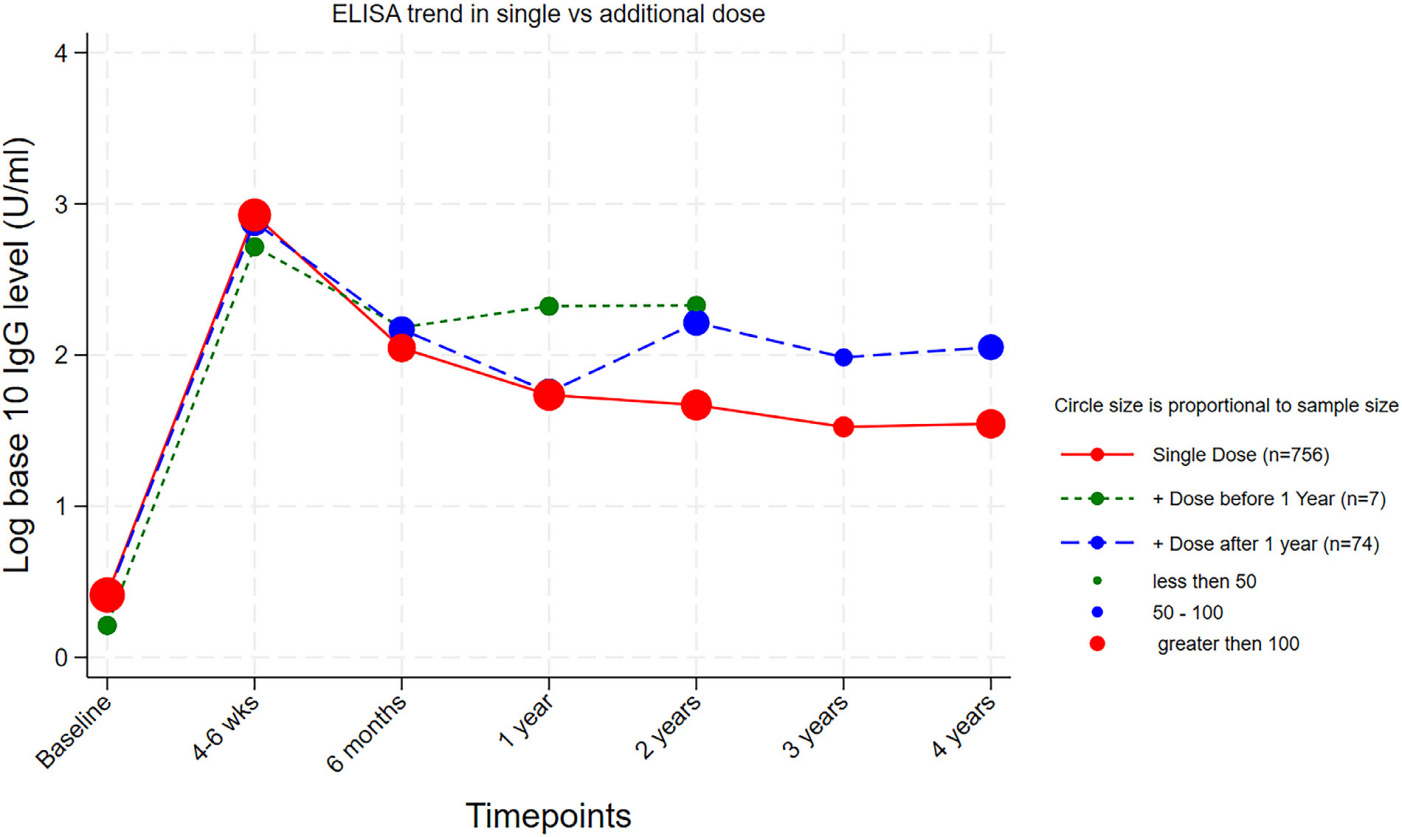
Immunoglobulin G levels of children who received a single vs two doses of Typbar TCV. ELISA, enzyme-linked immunosorbent assay; TCV, typhoid conjugate vaccine. Note: 14 children who received a second dose of TCV just within 1 week before the 1-year blood draw were included with those receiving their second dose after the 1-year time point.

Of the 81 children who received a second dose of TCV during the national campaign, 26% (21 of 81) received the second dose of TCV between 6 months to 1 year after the first dose. The median time to the second dose in these participants was 9.4 months (IQR: 9.4-9.4). A total of 60 children (74%) received the second dose of TCV between 1 to 2 years after the first dose. The median time to the second dose in these participants was 14.4 months (IQR: 12.0-16.4). A total of 99% (80 of 81) provided a blood sample for anti-Vi IgG ELISA at the 4-6–week time point and 88% (71 of 81) provided a blood sample for anti-Vi IgG ELISA at the 2-year time point. Seroconversion was observed in 77 of 80 (96.3%) of these children 4-6 weeks after their initial dose of TCV and in 65 of 71 (91.5%) at 2 years (Table S4). The anti-Vi IgG GMTs of the participants who received an additional dose of TCV are shown in Table S5. Three children failed to seroconvert even after receiving an additional dose before the 2-year time point. A higher sustained response at 2 years and beyond was observed in children who received a second dose of TCV dose (Figure 3).

## Discussion

In this long-term study of Pakistani children aged less than 10 years who were vaccinated with a single dose of TCV, we found that 95.8% successfully seroconverted at 4-6 weeks and 75.6% remained above the seroconversion threshold for 4 years, demonstrating a durable immunological response in a typhoid endemic setting. Antibody decay was more pronounced in the youngest children who were 6 months to 2 years of age at baseline; at 4 years after vaccination, nearly 40% in this subcohort had dropped below the seroconversion threshold. A total of nine breakthrough culture-confirmed typhoid cases were observed during the 4-year follow-up period; all were among children who had seroconverted at 4-6 weeks and most of the infections occurred more than 2 years after vaccination.

Our results are consistent with other studies reporting on immune response to TCV in endemic settings. Studies of TCV response in children in Bangladesh, Burkina Faso, and India also found seroconversion rates of 94-99.5% 4-6 weeks after vaccination [18,22,23]. A clinical trial in India found that 74.1% of participants remained seroconverted above baseline values at 2 years after vaccination [18]; in Burkina Faso, seroconversion remained above 88% 2.5 years after vaccination [24]; and in Malawi, sustained seroconversion at 2-3 years after vaccination was observed in 80% of the children [25].

In our study, we observed that a greater proportion of younger children seroconverted at 4-6 weeks than older children. In contrast, a study conducted in Nepal revealed almost equal seroconversion rate across all age groups (54 of 55, 98% in younger vs 225 of 225, 100% in older children) at day 28. This trend persisted even after 18 months of vaccination (55 of 58, 95% in younger vs 202 of 209, 97% in older children) [19]. Similarly, a study in India vaccinating with a different brand of TCV found no age differences in seroconversion, where 100% of children in all age strata seroconverted upon vaccination [26]. We observed that many participants who did not seroconvert at 4-6 weeks already had high antibody titers at baseline. Our cohort was drawn from a highly endemic environment and thus may have had a previous typhoid exposure. Recent data from a randomized, controlled trial in Malawi found 78.3% vaccine efficacy after 4 years. That same study found that vaccine efficacy fell by approximately 1.3% per year from the level of protection observed in the 1^st^ year of observation [27]. A similar decline in efficacy was observed in the Vi-rEPA two-dose vaccine trial. The efficacy was 93.9% during the first 12 months but declined to 89% by the end of the 46-month surveillance period [15,28].

A TCV immunogenicity sub-study conducted in Malawi observed higher anti-Vi IgG GMTs at day 28 among TCV recipients than seen in our study [25]. A similar finding was reported from India comparing two different brands of TCV, in which the overall GMT for the pediatric population (6 months to 18 years) at baseline was 5.7 EU/mL and rose to 1121.0 at 6 weeks follow-up after a single dose of TCV [29]. Another study from India noted higher GMTs at day 42 after TCV vaccination [18]. In the Vi-rEPA vaccine efficacy trial, the GMT at day 30 was high (87.42 µg/mL or 70.5 EU/mL) but declined to 4.8 µg/mL or 3.9 EU/mL over 46 months. A rapid rise and drop occurred during the 1^st^ year of vaccination, with levels reaching a steady state at later time points [28]. Although higher Vi IgG titers after vaccination have been found to reduce the probability of typhoid diagnosis, in the absence of a threshold of protection, it is not clear what antibody levels are necessary to maintain protective immunity [30].

Our study has reported a high proportion of sustained seroconversion with TCV vaccination in a setting endemic for XDR *S.* Typhi, where the prevalence of infection is high. Estimates of TCV vaccine effectiveness range from 79% to 85% in the 1^st^ year of vaccination and there is a possibility of breakthrough infections in 15-20% of vaccinated children [22,31,32]. Similar to a study in India [26], we observed few febrile episodes during the entire 4 years of active surveillance. However, we observed higher occurrence of culture proven *S*. Typhi infection; none of these children reported serious illness but four children required hospitalization. In contrast, data from Malawi reported a 1.3% incidence of the first episode of culture-confirmed *S*. Typhi infection over a surveillance period of 4.6 years, despite a higher frequency of febrile episodes [27]. The Malawi study team proactively educated parents to visit the study site when their children experienced a fever and monitored them actively; in our study, we relied on active surveillance via monthly phone follow-ups. In addition, the fear or stigma associated with reporting fevers during the pandemic or migrations may explain why we observed a lower frequency of reported fevers. In this study, we observed three deaths but none of them were due to typhoid fever.

Over a 4-year period, a significant decline in seroconversion was observed in younger children (aged 2 years or below) compared with the children above 2 years of age. Similar age-related differences in waning immunity were observed in the Malawi study, where lower seroconversion rates were noted in infants aged 9-11 months, whereas older children maintained higher seroconversion rates at 2-3 years after vaccination [23]. Similarly, Lahn *et al.* found that the levels of anti-Vi IgG antibodies induced in older children given a single dose of Vi-rEPA were similar to those in younger children who had received two doses [15]. Various environmental and genetic factors may play a role in the observed age-related variations. Younger children, particularly, those below 2 years of age, possess a less developed immune system and they may retain maternal antibodies for 6-12 months, thereby hindering their ability to mount a strong immune response during the 1^st^ year of life. Our results may have implications for optimizing the timing and dosing schedule of the vaccine in young children. However, there is no established correlate of protection for Vi; therefore, it is not known what antibody levels are required to protect against typhoid infection.

We were able to observe the impact of a second dose of TCV in children who received an additional dose of the vaccine during the government led mass immunization campaign. These children demonstrated a boosting response after the second dose of TCV and their titers remained elevated above children who received a single dose at 4 years after the initial dose. Longer-term follow-up is recommended to ascertain whether the protective efficacy of a single dose of the TCV persists for longer than 4 years after vaccination. Documenting the incidence of breakthrough infections during this follow-up period can help determine whether an additional vaccine dose is warranted.

Despite extensive follow-ups, not all participants could be contacted at all the time points of the study due to refusals; migration; COVID-19 pandemic lockdowns; and the addition of the 6-month, 3-year, and 4-year time points at a later stage in the study. Even after the lockdowns were lifted, the fear of COVID-19 and its associated stigma made follow-ups challenging because some parents not only refused to give blood samples but were also wary of reporting episodes of fever or other adverse events to the team. Consequently, we may have missed febrile episodes and enteric fever infections. Our study is conducting an ongoing analysis of anti-hemolysin E and *S.* Typhi lipopolysaccharide antibodies in the archived blood samples to investigate the prevalence of sub-clinical infection or missed diagnoses in the study population. Moreover, we had fewer participants at the 3-year time point due to delays in obtaining regulatory approvals. However, participants who were missed at the 3-year time point were re-approached and sampled at the 4-year time point.

Our study showed high rates of seroconversion after the administration of a single dose of TCV that was sustained over 4 years of follow-up in a highly endemic setting. However, we noted a steeper decline in antibody levels in the youngest children who were less than 2 years of age at vaccination. We also recorded a significant number of breakthrough infections occurring 2-3 years after vaccination. This study provides valuable observation of the immunogenicity and durability of response in children who received a second dose of vaccine. Although Pakistan has successfully added TCV to the Expanded Programme of Immunization schedule, continued surveillance and monitoring are essential to assess the population impact of routine TCV vaccination and explore the potential need to reevaluate the vaccination protocol, including the possible inclusion of booster doses.

## Declarations of competing interest

The authors have no competing interests to declare.

## Appendix Supplementary materials

Image, application 1
