# Supplementary material for: Longevity of immune response after a single dose of typhoid conjugate vaccine against Salmonella Typhi among children in Hyderabad, Pakistan
Source: Int J Infect Dis. 2024 Oct;147:None. doi: 10.1016/j.ijid.2024.107187 (PMC11442317; doi:10.1016/j.ijid.2024.107187)
Supplement: Supplementary file 1 [file mmc1.docx]

**Supplementary Tables**

**Table S1 (associated to Table 2): Pairwise comparison between age groups’ seroconversion.**

|  | **Seroconversion** | | | | | | | | **Pairwise comparisons between age groups seroconversion** | | |
| --- | --- | --- | --- | --- | --- | --- | --- | --- | --- | --- | --- |
|  | **Overall** | | **6M – 2 Years** | | **>2 – 5 Years** | | **>5-10 Years** | |  |  |  |
|  |  |  | **(A)** | | **(B)** | | **(C)** | | **(B) vs. (A)** | **(C) vs. (A)** | **(C) vs. (B)** |
| **Timepoints** | **n/N** | **%** | **n/N** | **%** | **n/N** | **%** | **n/N** | **%** | ***p-value** | ***p-value** | ***p-value** |
|  |  | **(95% CI)** |  | **(95% CI)** |  | **(95% CI)** |  | **(95% CI)** | **(95% CI)** | **(95% CI)** | **(95% CI)** |
| **Baseline** | 958 | ·· | 247 | ·· | 461 | ·· | 250 | ·· | ·· | ·· | ·· |
|  |  | ·· |  | ·· |  | ·· |  | ·· | ·· | ·· | ·· |
| **4-6 weeks** | 802/837 | 95·8 | 206/207 | 99·5 | 385/402 | 95·8 | 211/228 | 92·5 | 0·098 | 0·02 | 0·267 |
|  |  | (94·2, 97·0) |  | (96·6, 99·9) |  | (93·3, 97·4) |  | (88·3, 95·3) |  |  |  |
| **6 months** | 541/602 | 89·9 | 133/145 | 91·7 | 223/251 | 88·8 | 185/206 | 89·8 | 1 | 1 | 1 |
|  |  | (87·2, 92·0) |  | (86·0, 95·2) |  | (84·3, 92·2) |  | (84·9, 93·3) |  |  |  |
| **1 year** | 626/755 | 82·9 | 139/187 | 74·3 | 308/361 | 85·3 | 179/207 | 86·5 | 0·006 | 0·008 | 1 |
|  |  | (80·1, 85·4) |  | (67·6, 80·1) |  | (81·3, 88·6) |  | (81·1, 90·5) |  |  |  |
| **2 years** | 522/651 | 80·2 | 117/172 | 68·0 | 261/307 | 85·0 | 144/172 | 83·7 | <0·001 | 0·002 | 1 |
|  |  | (76·9, 83·1) |  | (60·7, 74·6) |  | (80·6, 88·6) |  | (77·4, 88·5) |  |  |  |
| **3 years** | 203/261 | 77·8 | 53/80 | 66·3 | 76/92 | 82·6 | 74/89 | 83·1 | 0·045 | 0·037 | 1 |
|  |  | (72·3, 82·4) |  | (55·2, 75·8) |  | (73·4, 89·1) |  | (73·9, 89·6) |  |  |  |
| **4 years** | 438/579 | 75·6 | 94/149 | 63·1 | 205/262 | 78·2 | 139/168 | 82·7 | 0·001 | 0 | 0·967 |
|  |  | (72·0, 79·0) |  | (55·0, 70·5) |  | (72·8, 82·8) |  | (76·2, 87·7) |  |  |  |

***Bonferroni method used for pairwise comparison**

**Table S2 (associated to Table 2): Pairwise comparison between age groups’ Geometric Mean titers·**

| **Timepoints** | **Geometric Mean Titers (95% CI) in various age groups** | | | | | | **Pairwise comparisons between  age groups GMTs** | | |
| --- | --- | --- | --- | --- | --- | --- | --- | --- | --- |
|  | **6M – 2 Years** | | **>2 – 5 Years** | | **>5-10 Years** | |  |  |  |
|  | **(A)** | | **(B)** | | **(C)** | | **(B) vs A** | **(C) vs (A)** | **(C) vs (B)** |
|  | **N** | **GMT** | **N** | **GMT** | **N** | **GMT** |  |  |  |
|  |  | **(95% CI)** |  | **(95% CI)** |  | **(95% CI)** |  |  |  |
| **Baseline** | 247 | 1·7 | 461 | 3·1 | 250 | 2·7 | <0·001 | 0·001 | 0·277 |
|  |  | (1·5 - 1·9) |  | (2·8 - 3·6) |  | (2·2, 3·2) |  |  |  |
| **4-6 weeks** | 207 | 662·0 | 402 | 958·4 | 228 | 800·1 | 0·001 | 0·217 | 0·156 |
|  |  | (564·1 - 776·8) |  | (861·3 - 1066·5) |  | (671·7, 952·9) |  |  |  |
| **6 months** | 145 | 57·8 | 251 | 135·9 | 206 | 152·9 | <0·001 | <0·001 | 0·494 |
|  |  | (45·9 - 72·9) |  | (120·8 - 152·9) |  | (132·4, 176·6) |  |  |  |
| **1 year** | 187 | 20·6 | 361 | 69·3 | 207 | 87·4 | <0·001 | <0·001 | 0·091 |
|  |  | (16·6 - 25·5) |  | (61·3 - 78·4) |  | (74·3 – 102·9) |  |  |  |
| **2 years** | 172 | 14·6 | 307 | 64·9 | 172 | 84·8 | <0·001 | <0·001 | 0·106 |
|  |  | (11·5 - 18·5) |  | (55·9 - 75·4) |  | (70·6 – 101·8) |  |  |  |
| **3 years** | 80 | 10·1 | 92 | 46·0 | 89 | 71·0 | <0·001 | <0·001 | 0·018 |
|  |  | (7·7 - 13·2) |  | (36·6 - 57·7) |  | (58·1, 86·8) |  |  |  |
| **4 years** | 149 | 12·6 | 262 | 40·1 | 168 | 71·1 | <0·001 | <0·001 | <0·001 |
|  |  | (9·8 - 16·3) |  | (34·4 - 46·6) |  | (59·5, 85·0) |  |  |  |

GMT= Geometric Mean titers·

**Table S3A: Demographic characteristics of culture confirmed enteric fever cases·**

| **Variables** | **Categories** | **N=10** |
| --- | --- | --- |
|  |  | **n (%)** |
| Age (Years), Median (IQR) |  | 3·6 (1·1-7·6) |
| Age | 6 mo-2 years | 4 (40·0) |
|  | > 2 – 5 years | 2 (20·0) |
|  | > 5 – 10 years | 4 (40·0) |
| Gender | Male | 6 (60·0) |
| Area of Residence | Qasimabad | 8 (80·0) |
|  | Latifabad | 2 (20·0) |
| Fathers Education | No formal education or religious education only | 4 (40·0) |
|  | Primary/Secondary | 1 (10·0) |
|  | Higher Secondary or above | 5 (50·0) |
| Mothers Education | No formal education or religious education only | 5 (50·0) |
|  | Primary/Secondary | 3 (30·0) |
|  | Higher Secondary or above | 2 (20·0) |
| Height (cm), Mean ± SD |  | 97·1 ± 24·0 |
| Weight (cm), Mean ± SD |  | 15·1 ± 6·7 |
| MUAC (cm), Mean ± SD |  | 15·7 ± 1·9 |
| BMI (Kg/m2), Mean ± SD (N=958) |  | 18·7 ± 7·3 |
| Nutritional status (N=855) | Well nourished | 6 (75·0) |
|  | (WHZ/WLZ or BAZ score ≥ -2 to ≤ +6) |  |
|  | Malnourished | 2 (25·0) |
|  | (WHZ/WLZ or BAZ score ≤ -6 to < -2) |  |

BMI= Body Mass Index· MUAC =Mid Upper Arm Circumference· WHZ= Weight for Height Z-score· WLZ= Weight for Length Z-score·

**Table S3B: Details of culture confirmed enteric fever cases.**

| S· No | Age at baseline | Age at illness | Gender | WHZ score/BMI Z-score | Date of TCV administration | Date of blood culture result | Time interval between vaccination and Infection | Dose(s) of TCV | Antibody Titers | Antibody Titers | Antibody Titers | Antibody Titers | Antibody Titers | Antibody Titers | Antibody Titers | Specie isolated |
| --- | --- | --- | --- | --- | --- | --- | --- | --- | --- | --- | --- | --- | --- | --- | --- | --- |
|  | (years) | (years) | F=Female |  | DD/MM/YYYY | DD/MM/YYYY | (years) |  | Baseline | 4-6 wks | 6 months | 1 year | 2 years | 3 years | 4 years |  |
|  |  |  | M=Male |  |  |  |  |  | U/ml | U/ml | U/ml | U/ml | U/ml | U/ml | U/ml |  |
| 1 | 2·5 | 3·2 | M | 2·72 | 22/11/2018 | 08/08/2019 | 0·7 | 2 | 1·31 | 1738·5 | 287·7^#^ | 58·9 | 233·7 | ·· | 130·5 | S· Paratyphi A |
| 2 | 1·1 | 2·4 | M | 2·42 | 17/11/2018 | 01/03/2020 | 1·3 | 1 | 5·07 | 521·3 | 114·9 | 11·9^#$^ | 10·2 | 12·9 | 8·1 | MDR-S· Typhi |
| ^*^ 3 | 6·7 | 9·1 | M | -2·24 | 19/12/2018 | 20/05/2021 | 2·4 | 1 | 0·63 | 468·2 | 31·6 | 64 | 19·5^#^ | 21·2 | 7·7 | XDR-S· Typhi |
| ^*^ 4 | 7·6 | 10·0 | M | -5·59 | 19/12/2018 | 24/05/2021 | 2·4 | 1 | 0·6 | 81·4 | 12·3 | 8·2 | 14·7^#^ | 13·3 | 10·9 | XDR-S· Typhi |
| ^*^ 5 | 9·1 | 11·6 | F | -4·53 | 19/12/2018 | 24/05/2021 | 2·4 | 1 | 0·74 | 388·7 | 146·2 | 145·8 | 136·6^#^ | 96·8 | 80·2 | XDR-S· Typhi |
| 6 | 0·7 | 3·3 | M | 5·46 | 27/11/2018 | 10/06/2021 | 2·5 | 1 | 1·2 | 417 | 15·1 | 3·1 | 4·2^#$^ | 3·9 | 4·6 | XDR-S· Typhi |
| 7 | 1·6 | 5·0 | F | 5·87 | 04/07/2018 | 19/12/2021 | 3·5 | 1 | 5·5 | 1300·9 | 80·5 | 8·4 | 7·57^#$^ | ·· | 26·2 | Non-MDR/XDR--S· Typhi |
| 8 | 9·3 | 13·0 | F | -2·12 | 11/07/2018 | 17/03/2022 | 3·7 | 1 | 1·3 | 2483·2 | 416·3 | 259·2 | 130·8^#^ | ·· | 159·4 | Non-MDR/XDR-S· Typhi |
| 9 | 4·8 | 8·5 | M | 0·11 | 12/07/2018 | 21/04/2022 | 3·8 | 1 | 3·0 | 2982·2 | 177·4 | 112·4 | 68·7^#^ | ·· | 62·4 | Non-MDR/XDR-S· Typhi |
| 10 | 1·1 | 4·6 | F | 6·34 | 22/11/2018 | 01/05/2022 | 3·4 | 1 | 148·2 | 1925·5 | 130·3 | 43·3 | 28·7 | 15·9^#$^ | 9·8 | MDR-S· Typhi |

*Cases are siblings

^#^Antibody titers prior getting infection (U/ml)

^$^Antibody titers fell below four-fold compared to baseline titers

(··) = Not applicable as sample not collected at this timepoint

**Table S4: Demographic characteristics of participants who received a second dose of Typbar-Typhoid Conjugate Vaccine·**

| **Variables** | **Categories** |  | **Baseline** |
| --- | --- | --- | --- |
|  |  |  | **N=81** |
|  |  |  | **n (%)** |
| Age (Years), Median (IQR) |  |  | 4·5 (3·0-7·0) |
| Age category | 6 mo-2 years |  | 10 (12·3) |
|  | >2 - 5 years |  | 37 (45·7) |
|  | >5 - 10 years |  | 34 (42·0) |
| Gender | Male |  | 44 (54·0) |
| Area of residence | Latifabad |  | 54 (66·7) |
|  | Qasimabad |  | 27 (33·3) |
| Height (cm), Mean ± SD |  |  | 100·3 ±16·5 |
| Weight (Kg), Mean ± SD |  |  | 15·6 ± 5·5 |
| MUAC (cm), Mean ± SD |  |  | 15·7 ± 2·3 |
| BMI (Kg/m^2^), Mean ± SD |  |  | 15·3 ± 2·8 |
| Nutritional status | WHZ score (N=44) | Normal (-1·99 to +6) | 42 (95·5) |
|  |  | Wasting (-2·99 to -2) | 1 (2·3) |
|  |  | Severely Wasting (-6 to -3) | 1 (2·3) |
|  | BAZ score (N=31) | Normal (-2<BAZ<+1) | 9 (29·0) |
|  |  | Underweight (< -2 SD) | 21 (68·0) |
|  |  | Overweight (+ 1 SD) | 1 (3·0) |
| Education of Father | No formal education or religious education only |  | 58 (23·5) |
|  | Primary/Secondary |  | 39 (48·1) |
|  | Higher Secondary or above |  | 23 (28·4) |
| Education of Mother | No formal education |  | 40 (49·4) |
|  | Primary/Secondary |  | 20 (24·7) |
|  | Higher Secondary or above |  | 21 (25·9) |
| Seroconversion at 4-6 weeks (N=80) |  |  | 77 (96·3) |
| Seroconversion at 2 years (N=71) |  |  | 65 (91·5) |

BMI= Body Mass Index· MUAC =Mid Upper Arm Circumference· WHZ= Weight for Height Z-score· BAZ= BMI for Age Z-score· (··) = Not applicable

**Table S5:** **Geometric Mean Titers (GMT) of participants who received an additional dose of TCV·**

|  | **Geometric Mean Antibody Titers (GMT) (U/ml)** | | | | | | | | |
| --- | --- | --- | --- | --- | --- | --- | --- | --- | --- |
| **Timepoints** |  |  |  |  |  |  |  |  |  |
|  | **Overall** | | **6M - 2** | | **> 2 - 5** | | **> 5 – 10** | | **p-value** |
|  |  |  | **Years** | | **Years** | | **Years** | |  |
|  | **N** | **GMT** | **N** | **GMT** | **N** | **GMT** | **N** | **GMT** |  |
|  |  | **(95% CI)** |  | **(95% CI)** |  | **(95% CI)** |  | **(95% CI)** |  |
| **Baseline** | 81 | 2·4 | 10 | 1·5 | 37 | 2·7 | 34 | 2·4 | 0·35 |
|  |  | (1·9 - 3·1) |  | (0·8 - 2·7) |  | (2·1 - 3·5) |  | (1·4 - 4·1) |  |
| **4-6 weeks** | 80 | 734·6 | 9 | 614·8 | 37 | 1056·3 | 34 | 518·6 | 0·017 |
|  |  | (577·6 - 934·3) |  | (341 - 1108·1) |  | (795·5 - 1402·6) |  | (334·4 - 804·4) |  |
| **6 months** | 69 | 150·7 | 7 | 98·8 | 30 | 175 | 32 | 143·6 | 0·36 |
|  |  | (119·1 - 190·5) |  | (41·9 - 232·6) |  | (131·5 - 232·9) |  | (95 - 217·2) |  |
| **1 year** | 76 | 66·2 | 9 | 52·1 | 33 | 70·7 | 34 | 66·1 | 0·85 |
|  |  | (48·1 - 91·1) |  | (13·6 - 199·2) |  | (45·2 - 110·5) |  | (39·6 - 110·3) |  |
| **2 years** | 71 | 161·9 | 8 | 75·4 | 30 | 193·2 | 33 | 165·9 | 0·097 |
|  |  | (124·7 - 210·1) |  | (15·7 - 362·6) |  | (131·2 - 284·7) |  | (122·6 - 224·5) |  |
| **3 years** | 26 | 96·6 | 2 | 121·6 | 6 | 114·3 | 18 | 89·1 | 0·72 |
|  |  | (71·4 - 130·6) |  | ·· |  | (45·7 - 285·8) |  | (62·7 - 126·6) |  |
| **4 years** | 60 | 112·5 | 6 | 89·3 | 29 | 113 | 25 | 118·2 | 0·76 |
|  |  | (90·8 - 139·3) |  | (30 - 266·4) |  | (80·6 - 158·5) |  | (87·8 - 159·2) |  |

Note: Age categories were selected from the enrollment timepoint, 81 children received a second dose of TCV during the national immunization campaign (the median time to second dose in 21 children was 9·4 months and in 60 children it was 14·4 months following the first dose).

**Figure S1: Comparison of GMT (Geometric Mean antibody Titers) in children who seroconverted versus those who did not seroconvert.**


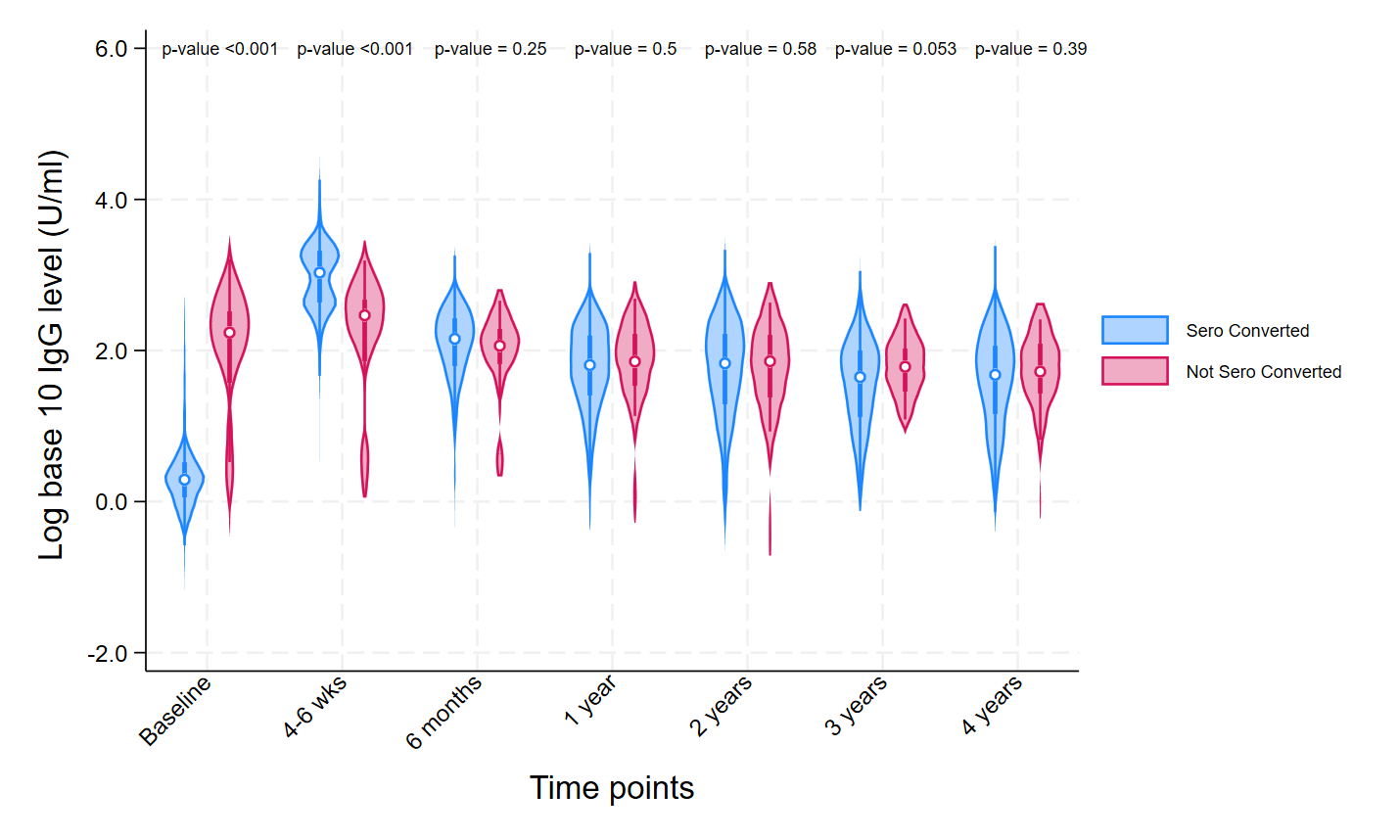


Independent sample t-test was applied to determine the association between median anti-Vi-IgG levels and seroconversion.
